# Analysis of differentially expressed genes in torn rotator cuff tendon tissues in diabetic patients through RNA-sequencing

**DOI:** 10.1186/s12891-023-07149-4

**Published:** 2024-01-03

**Authors:** Ziyang Yuan, Xu Zhu, Yike Dai, Lin Shi, Ziyang Feng, Zhiyao Li, Naicheng Diao, Ai Guo, Heyong Yin, Lifeng Ma

**Affiliations:** 1 Department of Orthopaedics, Beijing Friendship Hospital, Capital Medical University, Beijing, 100053 China; 2 Department of Orthopaedics, Beijing Lu He Hospital, Capital Medical University, Beijing, 101149 China

**Keywords:** Rotator cuff tear, Diabetes mellitus, RNA sequencing, Long non-coding RNA

## Abstract

**Background:**

Rotator cuff tears (RCT) is a common musculoskeletal disorder in the shoulder which cause pain and functional disability. Diabetes mellitus (DM) is characterized by impaired ability of producing or responding to insulin and has been reported to act as a risk factor of the progression of rotator cuff tendinopathy and tear. Long non-coding RNAs (lncRNAs) are involved in the development of various diseases, but little is known about their potential roles involved in RCT of diabetic patients.

**Methods:**

RNA-Sequencing (RNA-Seq) was used in this study to profile differentially expressed lncRNAs and mRNAs in RCT samples between 3 diabetic and 3 nondiabetic patients. Gene ontology (GO) and Kyoto encyclopedia of genes and genomes (KEGG) pathway analysis were performed to annotate the function of the differentially expressed genes (DEGs). LncRNA-mRNA co-expression network and competing endogenous RNA (ceRNA) network were constructed to elucidate the potential molecular mechanisms of DM affecting RCT.

**Results:**

In total, 505 lncRNAs and 388 mRNAs were detected to be differentially expressed in RCT samples between diabetic and nondiabetic patients. GO functional analysis indicated that related lncRNAs and mRNAs were involved in metabolic process, immune system process and others. KEGG pathway analysis indicated that related mRNAs were involved in ferroptosis, PI3K-Akt signaling pathway, Wnt signaling pathway, JAK-STAT signaling pathway and IL-17 signaling pathway and others. LncRNA-mRNA co-expression network was constructed, and ceRNA network showed the interaction of differentially expressed RNAs, comprising 5 lncRNAs, 2 mRNAs, and 142 miRNAs. TF regulation analysis revealed that STAT affected the progression of RCT by regulating the apoptosis pathway in diabetic patients.

**Conclusions:**

We preliminarily dissected the differential expression profile of lncRNAs and mRNAs in torn rotator cuff tendon between diabetic and nondiabetic patients. And the bioinformatic analysis suggested some important RNAs and signaling pathways regarding inflammation and apoptosis were involved in diabetic RCT. Our findings offer a new perspective on the association between DM and progression of RCT.

**Supplementary Information:**

The online version contains supplementary material available at 10.1186/s12891-023-07149-4.

## Introduction

Rotator cuff tears (RCT), usually caused by trauma and degeneration of the tendon tissue, is the leading cause of pain and functional disability of the shoulder [1]. The prevalence of RCT reaches to at least 10% among people over 60 years, which will leave a serious clinical problem in modern society [2]. There was a suggestion that surgical rotator cuff repair (RCR) had long-term improvement in shoulder functions for RCT [3], but not all patients’ outcomes of RCR are satisfactory [4] and re-rupture of rotator cuff after operation is not infrequent [5], which requires deep investigation of potential factors that may contribute to RCT. Diabetes mellitus (DM) is a common endocrine disease with an estimated prevalence of 9.3% worldwide in 2019, rising to 10.2% by 2030 and 10.9% by 2045 [6]. DM has been demonstrated to be strongly associated with increased risk of rotator cuff tendinopathy and RCT [7]. Noticeably, biological research has introduced the possible role of glucose as a risk factor for RCT [8], and recent evidence showed that the risk of rotator cuff diseases in the diabetic population is 2.11 times more than the non-diabetic population [9]. Additionally, patients with DM were 48% more likely to undergo RCR surgery compared to those without diabetes [10] and DM also impede rotator cuff healing with inferior outcome after rotator cuff repair. Notably, diabetic patients can achieve higher rate of healing after RCR with effective glycemic control [11]. Thus, it is critical important to understand the influence of DM on rotator cuff disease, which may provide deeper insight into the mechanism of RCT and its healing process.

It has been reported more than 3000 genes were differentially expressed between torn and RC tendon tissue by RNA sequencing (RNA-seq) technique [12]. RNA-seq analysis is a powerful tool to analyze the expression levels of all transcriptomes generated in the cells [13]. After total mRNA is sequenced and quantified, RNA-seq can describe the molecular mechanisms related to the pathogenesis of the disease [14]. Long non-coding RNAs (lncRNAs) are transcripts longer than 200 bp [15, 16] and have been reported to involved in biological process of tendon. For instance, Lu et al. [17] discovered that constant overexpression of lncRNA H19 promoted tenogenic differentiation in human tendon stem/progenitor cells (TSPCs), and also enhanced tendon repair in a mouse model. In addition, Ge et al. [18] profiled lncRNAs involved in rotator cuff tendinopathy in comparison with the normal tendon through RNA-Seq, and the results showed that 419 lncRNAs were statistically differentially expressed between 2 groups, which underlined the huge potential of lncRNAs in regulating the process of rotator cuff tendinopathy.

The purpose of this study is to profile the differently expressed mRNAs and lncRNAs in torn rotator cuff tendon between diabetic and nondiabetic patients and elucidate the potential roles of DM affecting RCT.

## Methods

### Patient enrollment, selection and clinical data

All the experiments and patients enrollment protocol were approved by Ethics Committee of Beijing Friendship Hospital, Capital Medical University. All surgeries were performed at the Beijing Friendship Hospital, and the samples were collected from April to August 2022. A total of 6 patients with rotator cuff tears in the supraspinatus who underwent arthroscopic rotator cuff repair were enrolled.

### Patient information

**Fig. 1:**
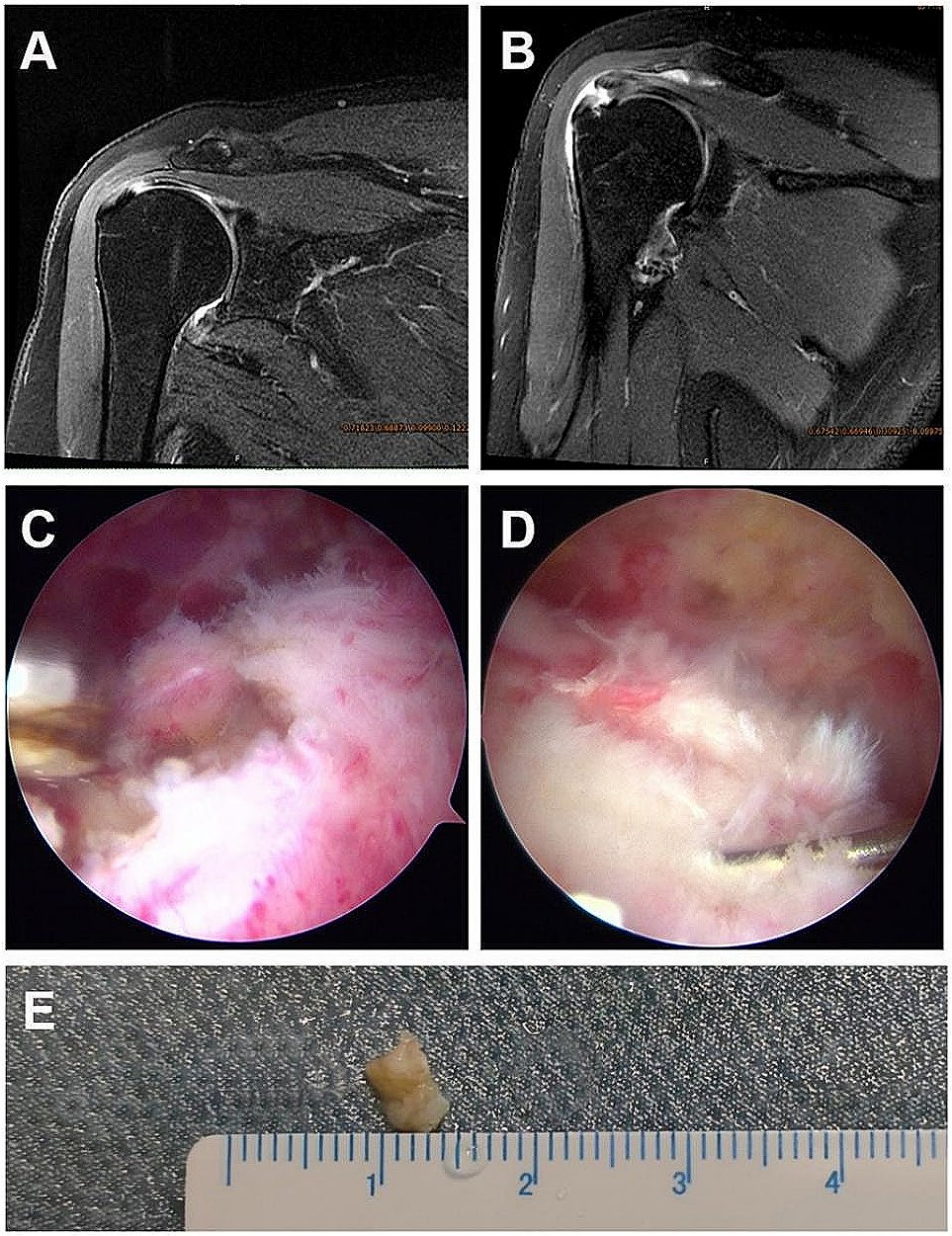
MRI images and arthroscopic view of torn rotator cuff in nondiabetic (**A**, **C**) and diabetic patients (**B**, **D**). **E** Macroscopic view of harvested tendon sample

Patients were enrolled in this study as a study group, who have diagnosed of type 2 DM (T2DM) after the age of 30 years without a history of ketosis. These non-diabetic patients were served as control group. The inclusion criteria for this study included: (1) full-thickness rotator cuff tear (1-3 cm); (2) arthroscopic rotator cuff repair was performed; (3) signed informed consent and voluntarily participated in the study. Exclusion criteria included: (1) previous history of systemic immunological diseases such as rheumatoid arthritis; (2) previous history of shoulder surgery and severe trauma; (3) In addition to rotator cuff repair, other procedures such as joint capsule repair and labrum repair were also performed on the ipsilateral shoulder. (4) patients unable to undergo magnetic resonance imaging (MRI) due to metal implants or claustrophobia; (5) irreparable rotator cuff tear larger than 5 cm; (6) severe cardiopulmonary dysfunction, history of peripheral nerve disease, peripheral vascular disease, renal insufficiency, and poorly controlled medical diseases; (7) The patient refused to participate in the study. In the end, a total of 6 patients who had rotator cuff tears in the supraspinatus underwent arthroscopic rotator cuff repair. All patients underwent preoperative MRI to be diagnosed with unilateral supraspinatus tears (Fig. 1A, B). All patients were arthroscopically operated by one experienced orthopedic surgeon (L.F.M.). A total of 12 patients were included in this study (6 diabetic RCT vs. 6 non-diabetic RCT), Among them, 6 RCT samples (n = 3 of each group) were used for RNA-seq; the other 6 samples (n = 3 of each group) were used for qPCR validation experiments. The detailed patient’s information was listed in supplementary Table S1.

### Tendon tissue harvesting

Rotator cuff tendon samples with a size of 3 × 3 mm were harvested from the edge of torn rotator cuff arthroscopically during operation (Fig. 1C–E). Samples were treated with RNAlater (Qiagen) immediately, froze in liquid nitrogen, and stored at − 80 °C for further RNA-Seq experiments.

### RNA isolation, library construction, sequencing, and qPCR validation

Total RNA was extracted using the Trizol reagent (Tiangen, Beijing, China) according to the manufacturer’s instructions. RNA concentration and purity was measured using the NanoDrop 2000 Spectrophotometer (Thermo Fisher Scientific, Wilmington, DE). RNA integrity was assessed using the RNA Nano 6000 Assay Kit of the Agilent Bioanalyzer 2100 System (Agilent Technologies, CA, USA).

As for the library construction, the Ribo-Zero rRNA Removal Kit (Epicentre, Madison, WI, USA) was used for rRNA removal. Sequencing libraries were constructed using NEBNextR Ultra^TM^ Directional RNA Library Prep Kit for IlluminaR (NEB, USA) following manufacturer’s recommendations, and index codes were added to attribute sequences to each sample. Fragmentation was carried out using divalent cations under elevated temperature in NEBNext First Strand Synthesis Reaction Buffer (5X). The all RNA was transcribed into double-stranded cDNA. Remaining overhangs were converted into blunt ends via exonuclease/polymerase activities. After adenylation of 3′ ends of DNA fragments, NEBNext Adaptor with hairpin loop structure were ligated to prepare for hybridization. The library fragments were purified with AMPure XP Beads (Beckman Coulter, Beverly, USA), to select insert fragments of preferentially 150 ~ 200 bp in length. Then 3 µl USER^TM^ Enzyme (NEB, USA) was used with size-selected, adaptor-ligated cDNA at 37° C for 15 min before PCR. Then PCR was performed with Phusion HighFidelity DNA polymerase, Universal PCR primers and Index(X) Primer. At last, PCR products were purified with AMPure XP Beads (Beckman Coulter, Brea, CA, USA), and library quality was assessed on the Agilent Bioanalyzer 2100 (Agilent Technologies, Santa Clara, CA, USA) and qPCR. The clustering of the index-coded samples was performed on acBot Cluster Generation System using TruSeq PE Cluster Kitv3-cBot-HS(Illumia) according to the manufacturer’s instructions. After cluster generation, the library preparations were sequenced by BioMarker Technologies (Beijing, China) on an Illumina platform (NovaSeq 6000) and reads were generated.

Quantitative PCR (qPCR) validation of four mRNAs (COL5, MMP2, EGR1, and EGR2) was using the iCycler iQTM Real-Time PCR Detection System (Bio-Rad). The relative gene expression was normalized to the glyceraldehyde 3-phosphate dehydrogenase (GAPDH) expression and is presented as the foldchange using the ΔΔ Ct method. The primers designed for qPCR was listed in supplementary Table S2.

### Data acquisition and bioinformatics analysis

Clean data (clean reads) were obtained by removing reads containing adapter, reads containing over 10% ploy-N and low-quality reads from raw data (raw reads). At the same time, Q20, Q30, GC-content and sequence duplication level of the clean data were calculated, to ensure the downstream analyses were based on clean data with high quality. Consequently, paired-end sequence files were mapped with reference genome hg38 using Hisat2 software. Gene abundance was visualized according to the fragments per kilobase of exon per million (FPKM) read. LncRNAs and mRNAs with FDR < 0.05 and |log2 (Fold Change) | ≥ 1 were assigned as differentially expressed. RT-qPCR validation was not used since RNA-seq methods are too robust to require validation by any other approaches [19].

Additionally, Gene Ontology (GO) analysis and Kyoto Encyclopedia of Genes and Genome (KEGG) analysis were used to determine the potential functions among these differentially expressed mRNAs [20].

### LncRNA-mRNA co-expression network analysis

Functional roles of the differentially expressed lncRNAs were predicted using the co-expression analysis. Basing on the standardized signal intensity of specifically lncRNAs and mRNAs, the co-expression network was established. LncRNA-mRNA with Pearson correlation coefficient value ≥ 0.9 along with P ≤ 0.05 were included. The lncRNAs-mRNA interaction network was visualized using Cytoscape software.

### LncRNA-miRNA-mRNA (ceRNA) network analysis

To identify interactions between differentially expressed mRNAs and lncRNAs, lncRNA-miRNA-mRNA networks were constructed and were visualized using Cytoscape software.

### Cis- and trans-regulation of lncRNAs

Based on the results of co-expression, differentially expressed lncRNAs were selected for cis- and trans-target gene prediction. Herewith, Cytoscape software (v3.9.0) was used to construct lncRNA-gene interaction networks, according to the results of differentially expressed lncRNAs and their corresponding differentially expressed cis- and trans-target genes.

### Potential transcription factors target of lncRNA

According to the gene co-expression results, the transcription factors (TFs) were searched for these associated with lncRNAs to explore their potential roles in RCT. Predict TF and Predict TFBS software were used to predict TFs via AnimalTFDB database.

## Results

### Expression profiles of mRNAs and lncRNAs

**Fig. 2:**
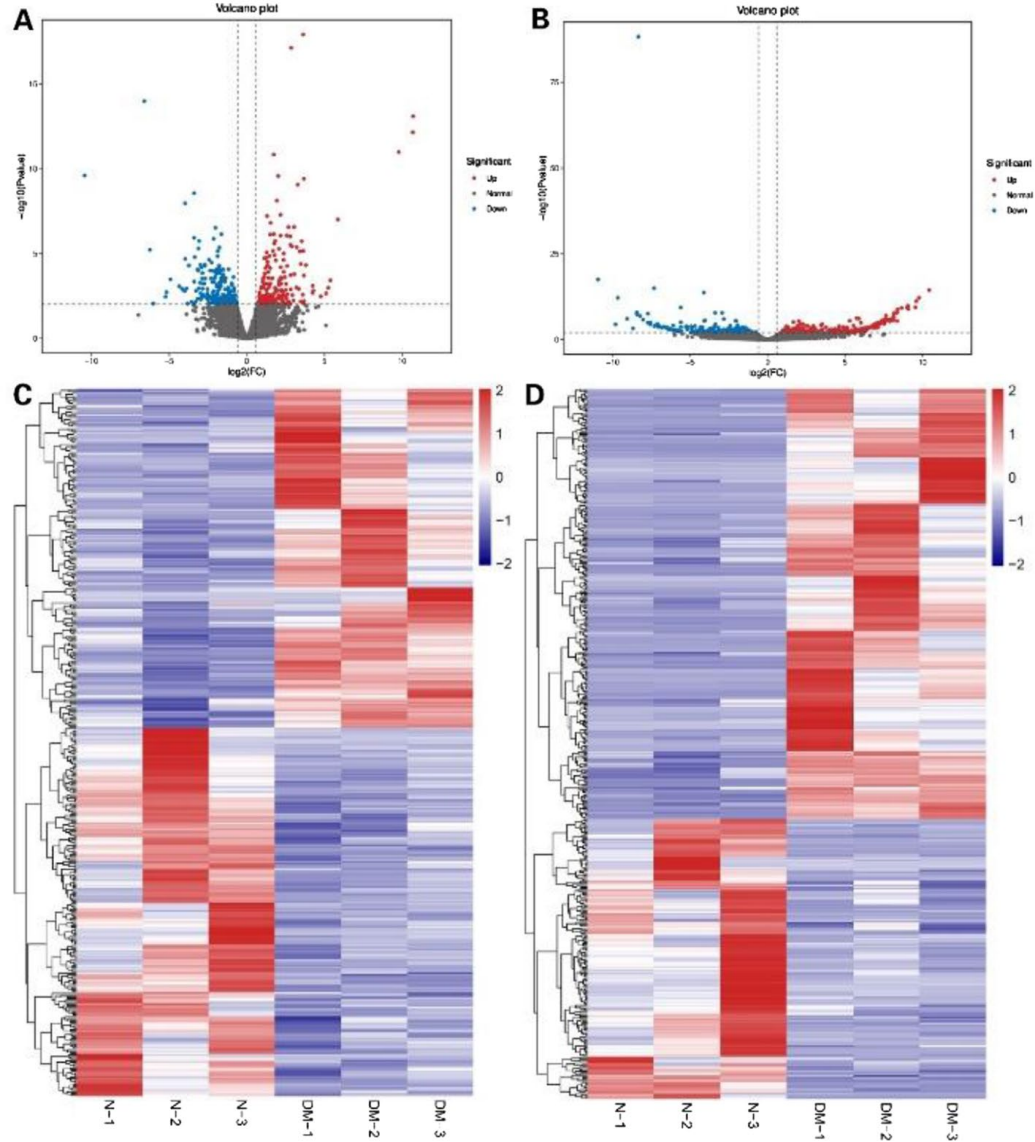
Volcano plots (**A**, **B**) and heat maps (**C**, **D**) showing expression profiles of lncRNAs and mRNAs between diabetic and nondiabetic patients. **A** Volcano plot of the differentially expressed lncRNAs. Red points denote up-regulated mRNAs, and blue points denote down-regulated mRNAs. **B** Volcano plot of the differentially expressed mRNAs. **C** Heatmap depicting expression levels of the lncRNAs. The red stand for the up-regulated DEGs and the blue stand for down-regulated DEGs. **D** Heatmap depicting expression levels of the differentially expressed mRNAs

Based on the whole expression profile, we identified 29,610 lncRNAs and 505 differentially expressed lncRNAs, including 306 upregulated lncRNAs (such as HAGLR-207, MSTRG.157426.1 and others) and 199 down-regulated ones (MSTRG.166890.1, MSTRG.12914.1 and others) in Fig. 2A. 186 down-regulated mRNAs (MMP11, ADAM19 and others) and 202 up-regulated mRNAs (such as TSPOAP1, HS3ST1 and others) were detected from 388 differentially expressed mRNAs (Fig. 2B). Heatmaps of the differentially expressed lncRNAs and mRNAs are presented in Fig. 2C, D. The differentially expressed mRNAs and lncRNAs have been listed in supplementary Tables S3 and S4. Among those differentiation expressed mRNAs, several thoroughly studied molecules including COL5, MMP2, EGR1, and EGR2 were downregulated in the diabetic RCT samples. We further performed qPCR validation of these mRNAs, and the results were consistent with RNA-seq (Supplementary Fig. S1).

### Gene ontology (GO) and Kyoto encyclopedia of genes and genomes (KEGG) enrichment analysis

**Fig. 3:**
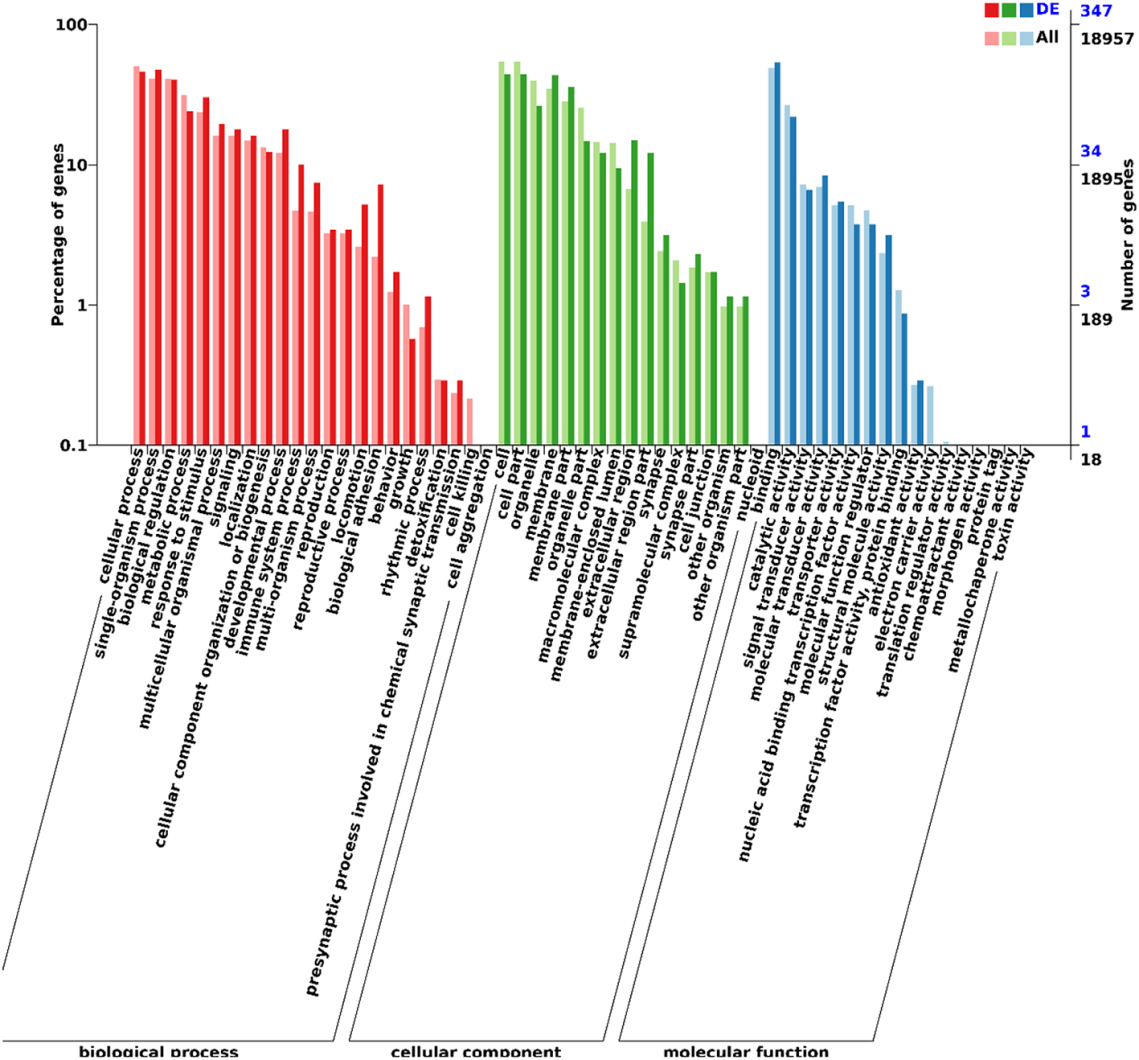
Gene Ontology (GO) enrichment analysis for the differentially expressed mRNAs, in terms of biological process (red), cellular component (green) and molecular function (blue)

The function of DEGs was described by GO analysis to gain deeper insights with terms involved in biological process, cellular component and molecular function. The DEGs involved in the biological process were mainly related to metabolic process (annotated by 84 DEGs) and immune system process (annotated by 35 DEGs). The DEGs involved in the cellular component were mainly related to cell and cell part (both annotated by 154 DEGs). The DEGs involved in molecular function were mainly related to binding (annotated by 187 DEGs) and catalytic activity (annotated by 76 DEGs) (Fig. 3).

**Fig. 4:**
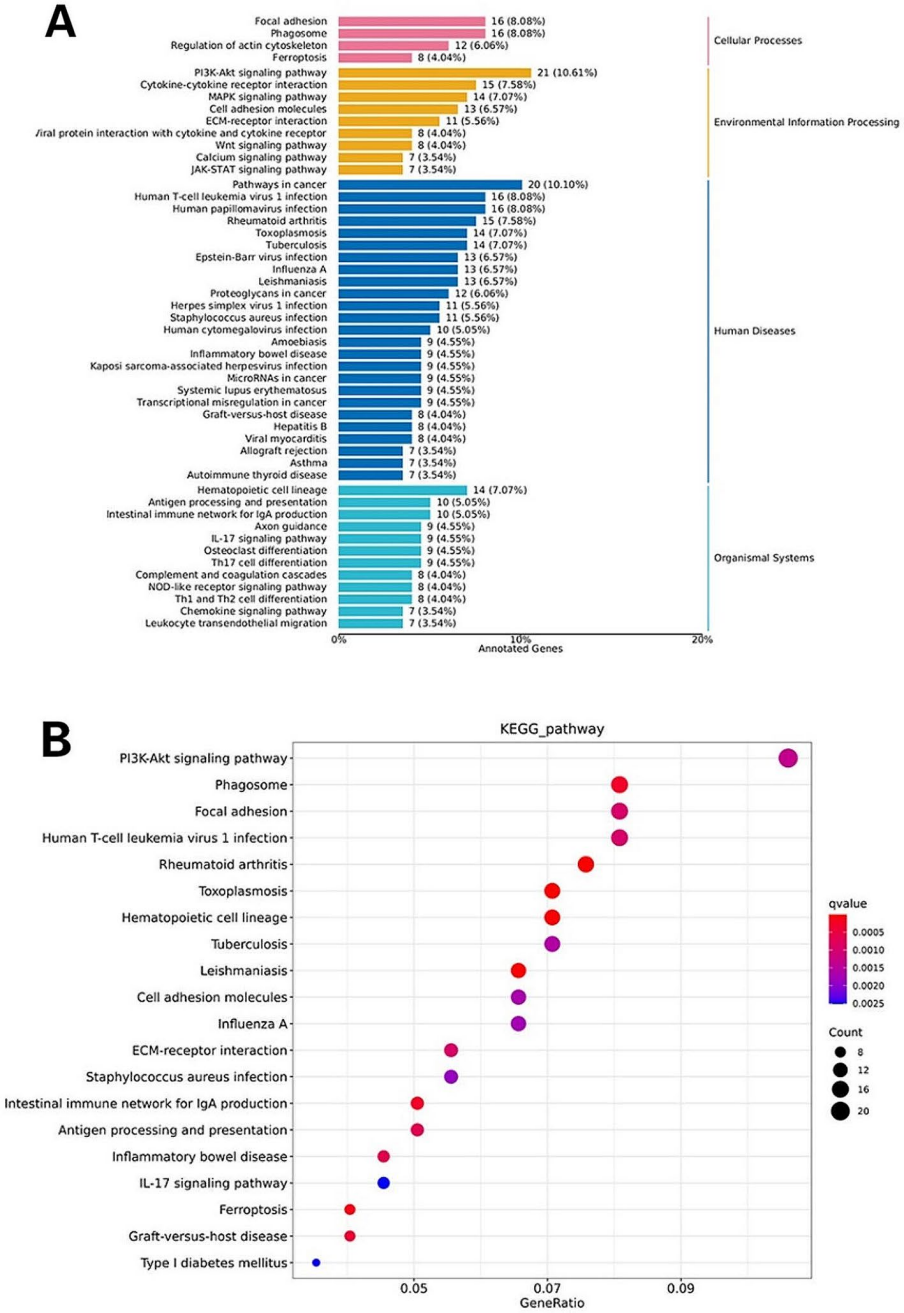
Kyoto encyclopedia of genes and genomes (KEGG) pathway enrichment analysis scores of up-and down regulated lncRNAs and mRNAs. **A** KEGG classification of the differentially expressed mRNAs. X-axis label represented annotated genes; Y-axis label represented pathway. **B** KEGG enrichment of the differentially expressed mRNAs. The x-axis called gene ratio represented the enrichment degree and the y-axis represented pathway

As for the KEGG analysis, which was conducted to predict the pathways of DEGs, the KEGG pathways comprised cellular processes, environmental information processing, genetic information processing, human diseases, metabolism, and organismal systems. The differentially expressed mRNAs were enriched in the ferroptosis (about 8 DEGs, such as CP and TFRC), PI3K-Akt signaling pathway (about 21 DEGs, such as COMP and IL-6), Wnt signaling pathway (about 8 DEGs, such as NFATC1 and MYC), JAK-STAT signaling pathway (about 7 DEGs, such as MCL1 and IL-7R) and IL-17 signaling pathway (about 9 DEGs, such as MMP9 and IL-6) (Fig. 4A, B).

### Co‑expression network of lncRNA‑mRNA

**Fig. 5:**
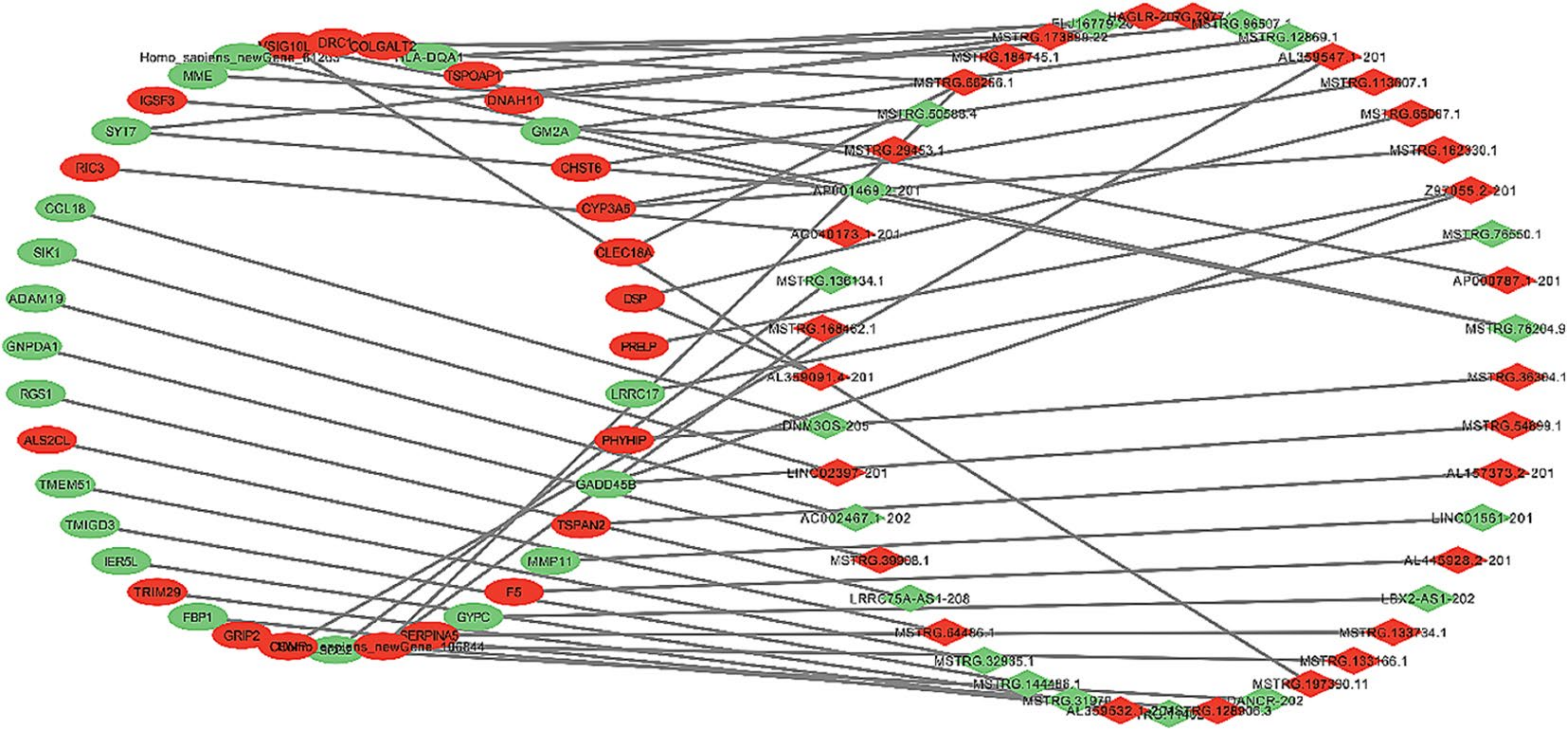
The lncRNA-mRNA co-expression network suggested an inter-regulation of lncRNAs and mRNAs. The rhombuses denote lncRNAs and the ellipses denote mRNAs (green: downregulated genes; red: upregulated genes)

To identify potential functions of the identified lncRNAs and mRNAs, we structured the lncRNA-mRNA co-expression network (Fig. 5). 15,221 lncRNA-mRNA pairs with significant Pearson correlation coefficient values (p < 0.05) were selected. In addition, the top 50 associations (ranking with p value) were contributed to the network diagram containing 47 remarkable expressed lncRNAs and 38 remarkable expressed mRNAs such as MMP11 and TSPOAP1. This network showed the overall prospect of the complex regulatory relationship among lncRNA and mRNA in RCT patients with DM. In this network, different lncRNAs can regulate one mRNA, and meanwhile specific lncRNA can regulate various of mRNAs, which constructed a complicated regulatory mechanism.

### ceRNA network analysis

**Fig. 6:**
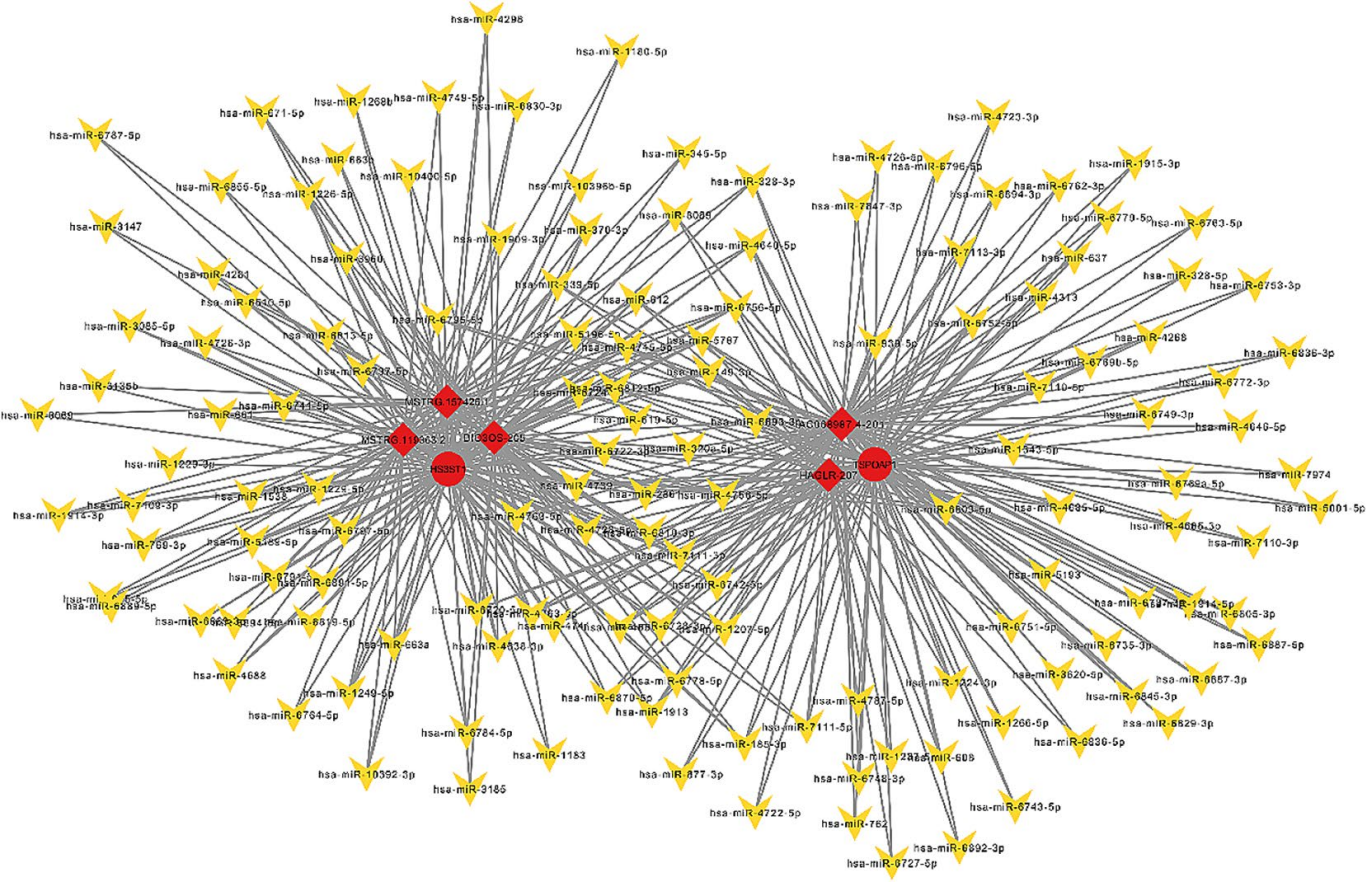
LncRNA-miRNA-mRNA (ceRNA) regulatory network. LncRNAs, miRNAs, and mRNAs were presented as quadrilateral, arrowheads and round, respectively (red: upregulated genes). LncRNA, long non-coding RNA; ceRNA, competing endogenous RNA

Based on the competing endogenous RNA (ceRNA) hypothesis, lncRNAs can regulate the expression of mRNAs through acting as molecular sponges of miRNAs [1]. A series of studies have explored the lncRNAs-miRNAs-mRNAs interactions in RCT [1, 18]. In this study, a ceRNA network was constructed to investigate potential interactions among lncRNAs, mRNAs, and miRNAs (Fig. 6). Basically, we selected top 5 lncRNAs (ranking with FDR), 2 mRNAs and 142 miRNAs. The most-linked lncRNA was AC068987.4-201 and had 74 edges, the most-linked mRNA was TSPOAP1 with 89 edges, and the most-linked miRNA, hsa-miR-5787, had 7 edges.

### Cis and trans targets of lncRNAs

**Fig. 7:**
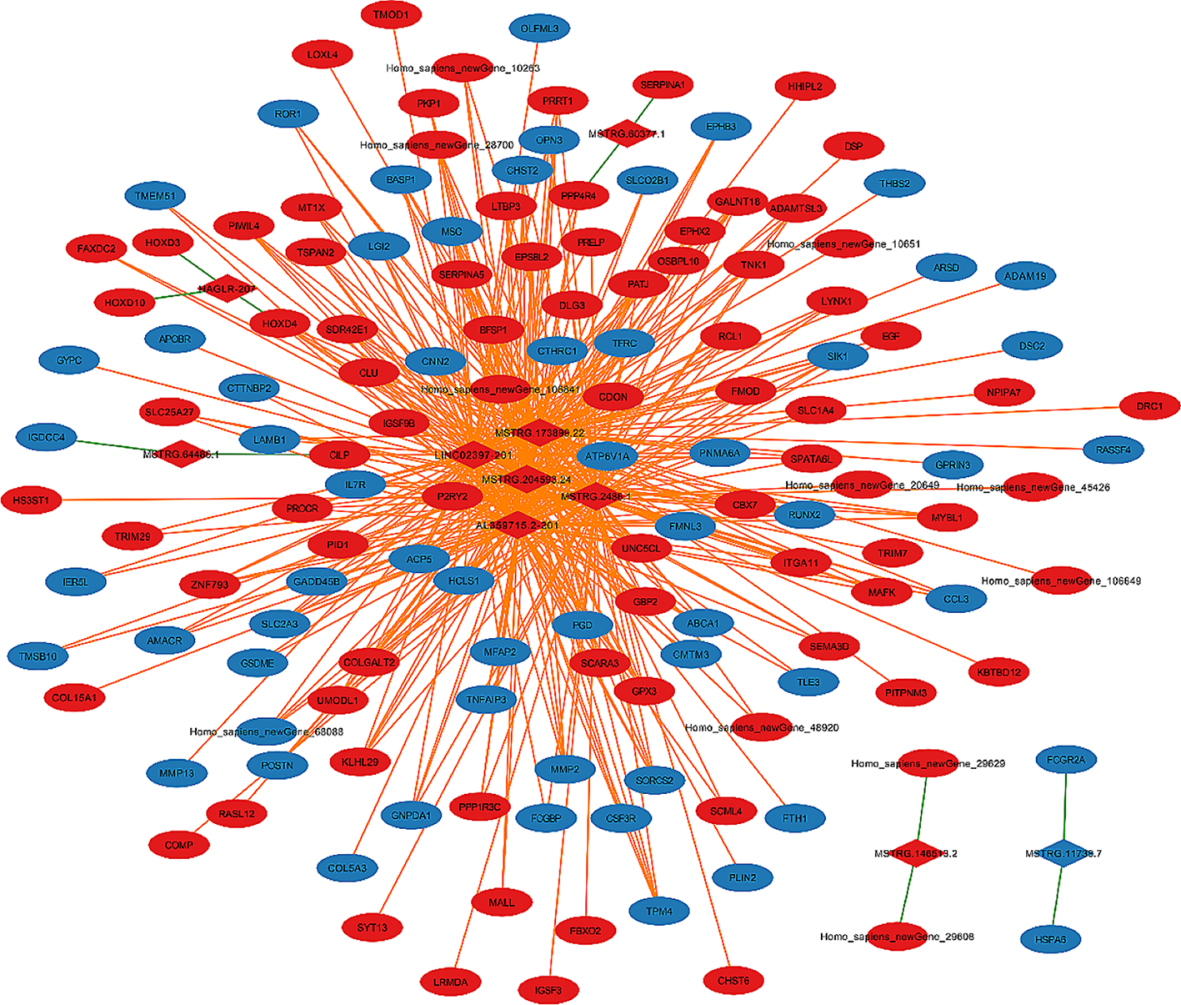
Networks analysis of interactions between selected lncRNAs and cis- and trans-regulated mRNAs related to the RCT. The rhombuses denote lncRNAs and the ellipses denote mRNAs (green: downregulated genes; red: upregulated genes). Orange lines indicate trans target genes and green lines indicate cis target genes, respectively

As shown in Fig. 7, the prediction of the potential cis- and trans- targets of lncRNAs was to dig the functions of Top 5 differentially expressed lncRNAs with the largest number of targets. As a result, 5 lncRNAs had 11 cis-regulatory mRNAs, and 5 lncRNAs had 407 trans-regulatory mRNAs, respectively. Interestingly, the interactive networks are quite complicated since one lncRNA (such as MSTRG.11738.7) can target many mRNAs (FCGR2A and HSPA6). These results provide valuable clues to the potential regulatory mechanisms of these differentially expressed lncRNAs in RCT.

### TFs role of lncRNAs

**Fig. 8:**
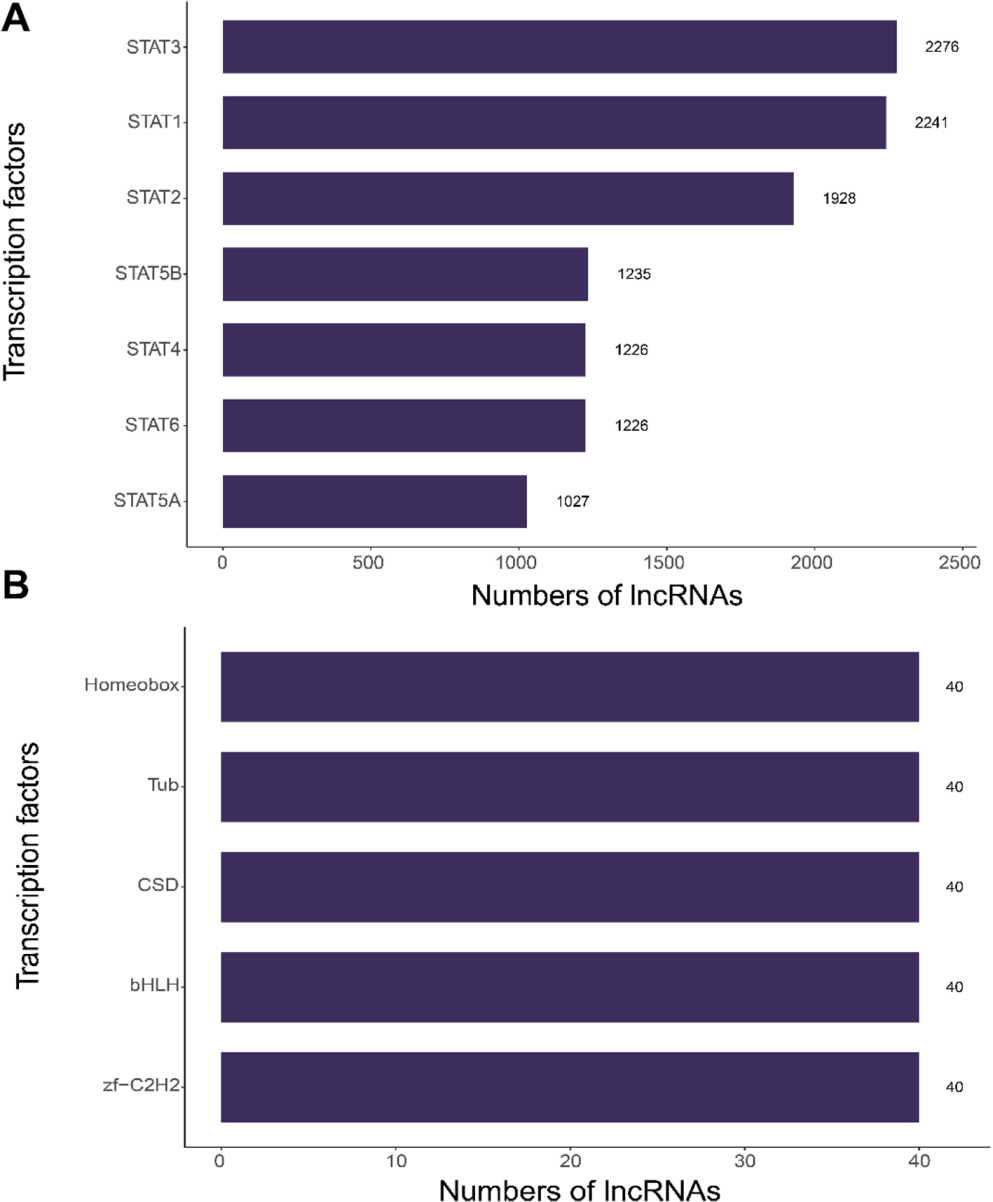
lncRNA–TF core network analysis. **A** Top 200 pairs of lncRNAs and TFs according to the Pearson correlation coefficient. **B** LncRNAs-STAT with Pearson correlation coefficient value ≥ 0.8 and p value ≤ 0.05

We predicted the potential TF targets of Top 200 differentially expressed lncRNAs, according to the Pearson correlation coefficient, to dig their functions in RCT. Hence, a total of 200 lncRNA–TF pairs were found, corresponding to 200 TFs (Fig. 8A). Besides, based on our fundings in KEGG analysis, we selected STAT, enriched by differentially expressed lncRNAs, to draw the column chart and to do further analysis (Fig. 8B).

## Discussion

Rotator cuff, consisted of the supraspinatus, infraspinatus, teres minor and subscapularis muscles, is recognized as pivotal role in providing dynamic stabilization of the glenohumeral joint as well as contributing to shoulder movement [21]. And RCT, especially degenerative tears are far more frequent and multifactorial in etiology [22]. A series of factors have been evaluated to be risk for progression of RCT, including smoking, location and age [23]. Correspondingly, it has been reported that RCT is most prevalent in the middle-aged and older adults and becomes the primary reason for shoulder surgery [24]. Recently, there is a growing awareness that rotator cuff tendinopathy is highly prevalent in diabetic patients [25, 26], and the absence of DM was found to be associated with better successful recovery after arthroscopic repair of RCT [27]. Bedi et al. [28] showed that diabetic rats had significantly less fibrocartilage after repair of RCT in contrast with nondiabetic animals. Besides, less organized collagen at the tendon-bone interface, as well as greater advanced glycosylation end products, was obviously observed in the diabetic group, which demonstrated sustained hyperglycemia impaired tendon-bone healing postoperatively. Similarly, Chung believed that inferior outcome of rotator cuff healing in patients with DM might be explained by impaired collagen production and collagen matrix formation, accumulation of advanced glycation end products [29]. Besides, the hyperglycemic condition potentially promoted poor healing of rotator cuff because of inadequate production of several important cytokines or growth factors [29]. As for the gene expression profiling of patients with RCT, Ren et al. [30] identified the differentially expressed lncRNAs in inflammatory long head of biceps tendon (LHBT), which may cause chronic RCT. Based on their results, they found that lncRNA‑COL6A4P2, A2MP1 and LOC100996671 may act as a regulator in the process of the inflammation of LHBT in RCT patients through NFKB2/NF‑kappa B signaling pathway. Furthermore, Zhang et al. [1] comprehensively dissected the dysregulated transcriptome of RCT, including mRNAs, miRNAs, lncRNAs, and circRNAs. They constructed the lncRNA/circRNA-associated dysregulated ceRNA networks and identified several important ncRNAs among it (hsa_circ_0000722, hsa-miR-129-5p and hsa-miR-30c-5p). However, there haven’t been such a study to explore the role of DM in RCT by using RNA-Seq. To our knowledge, this is the first study to present a systematic dissection of the differential expression profile of mRNAs and lncRNAs in RCT between diabetic and non-patients and revealed some important functional enrichment pathways which may contribute to the development of diabetic RCT.

In the current study, we successfully obtained the transcripts that were dysregulated in diabetic patients with RCT through identifying differentially expressed lncRNAs and mRNAs, compared with nondiabetic patients. The GO and KEGG pathway enrichment analyses indicated that several pathways were potentially associated with DM in RCT. GO analysis was applied to annotate the biological processes of DEGs, the results showed that they were related to metabolic process and immune system process. It is unsurprising that enrichment analysis of these dysregulated lncRNAs and mRNAs was associated with metabolic process in diabetic patients. Correspondingly, PI3K/AKT pathway has been reported to be identified as therapeutic targets of obesity and T2DM [31].

Collagen is the main components in the native tendon matrix. Our results demonstrated that the mRNA expression of collagen V and collagen X were downregulated in the diabetic RC tendon tissue. Collagen V is expressed in native tendon tissue and plays a critical regulatory role in maintaining normal tendon structure and function [32]. It was reported that collagen V knockdown impacted collagen fibril size and shape during tendon healing [33]. In addition, the expression of collagen X is found in the fibrocartilage of tendon-to-bone interface and persists through maturity and plays a role in the tissue mineralization [34]. Taken together, the down regulation of collagen V and collagen X in the diabetic RC tendon supports the in vivo histological results of significantly less fibrocartilage and organized collagen at the tendon-bone interface within the healing enthesis of diabetic animals [28].

Early growth response 1 and 2 (Egr1/2) are important transcription factors act as molecular sensors for guiding the final steps of tendon maturation and production of collagens and proteoglycans [35]. It has been proved that Egr1 and2 play a critical role in directing tenogenic differentiation and promoting tendon repair [36]. Tao ta al. reported that Egr-1 induced tenogenic differentiation of tendon-derived stem cells and enhanced rotator cuff repair after injury in a rabbit model [37]. In the current study, both Egr1 and Egr2 were down-regulated in the diabetic RCT samples. This finding is consistent with the results reported by Wu et al. [38]. In their study, results indicated that high glucose alters tendon homeostasis through downregulation of the AMPK/Egr1 pathway.

The role of immune inflammatory response in RCT should also be paid attention. Based on Chung et al. [29] study, increased glucose level could lead to induce inflammatory cytokines in torn rotator cuff tendon tissue of diabetic patients, especially MMP9 and IL-6. However, Lewandowsk et al. [39]reported that the concentrations of MMP-2 and MMP-9 were lower in subjects with type 2 DM than in non-diabetic controls. In the current study, MMP-2 and MMP-11 were found to be down-regulated in the diabetic RC tendon. Based on the lncRNA-mRNA co-expression network analysis, MMP11, which highly correlated with LINC01561-201, has been reported to strikingly protect against T2DM, while MMP11 deficient mice presented hallmarks of metabolic syndrome [40]. It was relevant to our data since MMP11 was apparently downregulated in diabetic patients. Viewed from above, regulation of MMPs appears to be complex in diabetic conditions, further deeper researches need to be carried out to decipher the role of MMPs in the pathological process of diabetic rotator cuff tear. In addition, our results indicated an up-regulation of IL-16, which is a pro-inflammatory pleiotropic cytokine in the diabetic group. Refer to literature, IL-16 gene polymorphism was correlated with type 2 DM [41].

The KEGG pathway analysis showed that DEGs were highly enriched IL-17 signaling pathway. In fact, IL-17 A is a pro-inflammatory cytokine and has been shown to be upregulated in early human tendinopathy. Mimpen et al. [42] demonstrated that IL-17 A and its receptors were present in torn supraspinatus tendon tissue. Besides, they treated tendon-derived fibroblasts with IL-17 cytokines and confirmed that they induced a direct response and activated diverse pro-inflammatory signaling pathways, which indicated the IL-17 acted as amplifiers of tendon inflammation and should be target as potential therapeutic role in tendinopathy. Millar et al. [43] found that the expression of IL-17 A was increased in early tendinopathy. Tenocytes treated with IL-17 A presented a series of changes including increased type III collagen. It has been reported that the proportion of type III collagen would be increased in RCT [44], and the increasing expression ratio of type-III to type-I collagen can affect the biomechanical properties of rotator cuff tendons [45]. These findings indicate that IL-17 signaling pathway may be a feature in diabetic RCT and can be conducive to the occurrence of RCT. Furthermore, the role of ferroptosis, which was enriched in this study, in tendon injury has been investigated, since Wu et al. [46] used its inducer RSL3 to inhibit the tenogenesis in vitro and in vivo. As for Wnt signaling pathway, Chen et al. [47] found that enhanced expression of Wnt5a in aged TSPCs caused canonical to noncanonical Wnt signaling shift, and they demonstrated that Wnt5a regulated TSPCs senescence via JAK-STAT signaling pathway, which was also involved in KEGG analysis. Conversely, genetic knockdown of JAK2 or STAT3 strongly alleviated TSPCs senescence of aged TSPCs [48]. Therefore, we screened these, enriched pathways that were involved in complicated process related to tendon disorders or inferior properties of tendon. Taken together, these bioinformatically predicted signal pathways interfered by these lncRNAs could serve as a reference for future studies of diabetic RCT and should be validated in further experiments.

TSPOAP1 was strongly linked with HAGLR-207 and was also the most-linked mRNA in ceRNA network. In fact, it mediates the inflammatory feedback through TNFR1 and downstream NF-κB, a transcription factor that promotes inflammation [49] and has been reported to involve in tendinopathic and ruptured Achilles tendon [50]. Furthermore, it plays a central role in inflammation by modulating the response of NLRP3 inflammasome, which is induced by TLR ligands, such as lipopolysaccharide via NF-κB signaling [49]. Since it was upregulated in diabetic RCT patients, investigating the role of TSPOAP1 mediated inflammation could provide new directions for in-depth studies of DM affecting RCT.

Zhang et al. [1] has reported that several miRNAs, such as hsa-miR-129-5p and hsa-miR-30c-5p which were dysregulated in inflammation-related diseases, may involve in inflammatory response in RCT. In our study, hsa-miR-5787 was one of the miRNAs identified in ceRNA network. And it was reported to involve in the process of glucose metabolism [51]. Meanwhile, it can attenuate LPS/TLR4-mediated inflammatory response via NF-κB in ischemic cerebral infarction [52]. We also found that one of its target mRNAs, HS3ST1, was reported to regulate Glucose-induced insulin secretion [53], and HS3ST1-/- mice presented a strong proinflammatory phenotype that was unresponsive to anti-inflammatory activity of plasma antithrombin [54], which indicated that hsa-miR-5787 play an important role in regulating inflammatory responses in diabetic RCT patients.

We also predicted cis- and trans- targets of Top 5 differentially expressed lncRNAs and found that the function of lncRNAs was complicated, since one lncRNA (such as MSTRG.204593.24) can target many mRNAs, and some mRNAs (such as KLHL29) can be regulated by various lncRNAs. In fact, different regions of human rotator cuff tendon specimens have variable balance between apoptotic and inflammatory processes, which is controlled by pro-and anti-apoptotic mechanisms and signals [55]. As for TFs, we found that the STAT protein family members may play important roles in RCT development, trans-regulated by differentially expressed lncRNA in diabetic patients. Importantly, STAT proteins can mediate apoptosis through a variety of pathways, mainly due to transcriptional activation of genes that mediate or trigger the cell death process (such as Bcl-xL, caspases, Fas and TRAIL) [56]. Significantly, high glucose has been demonstrated to induce cell apoptosis and suppress the tendon-related markers expression of tendon-derived stem cells in vitro [57]. And the high level of apoptosis in diabetic patients might impede tendon repair after injury [58]. Furthermore, the impact of apoptosis in rotator cuff disease has also been investigated. Yuan et al. [59] found that the number of cells undergoing apoptosis, which are mainly fibroblast-like cells, in the torn edges of rotator cuff tendons is twice more than that of normal tendons. Interestingly, the formation of advanced glycation end products, caused by DM, may promote cellular apoptosis in tendons via the expression of pro-apoptotic cytokine [60]. In conclusion, differentially expressed lncRNAs trans-regulate STAT in diabetic patients, thereby mediating apoptosis pathway and affecting the progression of RCT. Crucially, each member of STAT protein family has a unique role in apoptosis, since STAT1 activation is pro-apoptotic, but conversely, STAT 5 promotes cell survival, whereas STAT3 activation can have positively or negatively regulates cell survival, which depends on the stimulus and cell type [56]. Hence, the specific role of STAT proteins in regulating the apoptosis pathway and thus affecting the progression of RCT needs further investigation.

Albeit we first systematically profile the differentially expressed lncRNA and mRNA in RCT between diabetic and nondiabetic patients and identified some signaling pathways as well as the potential mechanism, this study still has several limitations. First, this study is based on a relatively small sample size of three pairs of torn supraspinatus tendon samples from patients with/without DM, which may have limited generalizability and cause the possibility of presenting false negative results in some genes. Addition to this, the precise mechanism of how DM affecting RCT is not deciphered. Future trials with larger sample size and in-depth molecular experiments are needed to be carried out to reveal the precise molecular association between DM and RCT.

## Conclusion

In summary, we first constructed and analyzed the differential expression patterns of lncRNAs and mRNAs in diabetic and nondiabetic RCT patients. Bioinformatic analysis suggested some signaling pathways regarding inflammation and apoptosis were involved in diabetic RCT. Our findings offer a new perspective on the association between DM and progression of RCT. Further in-depth molecular experiments are still demanded to validate our findings decipher the underlying precise molecular mechanism.

### Electronic supplementary material

Below is the link to the electronic supplementary material.

**Supplementary Material 1**. Summary of patient information

**Supplementary Material 2**. Real-time quantitative PCR primer sequences

**Supplementary Material 3**. Differentially expressed mRNAs

**Supplementary Material 4**. Differentially expressed LncRNAs

**Supplementary Material 4**. QPCR validation of differentially expressed mRNAs

## Data Availability

The datasets generated and/or analyzed during the current study are available in the Gene Expression Omnibus (GEO) repository, accession number GSE236746 (https://www.ncbi.nlm.nih.gov/geo/).
