# Supplementary material for: Analysis of differentially expressed genes in torn rotator cuff tendon tissues in diabetic patients through RNA-sequencing
Source: BMC Musculoskelet Disord. 2024 Jan 3;25:31. doi: 10.1186/s12891-023-07149-4 (PMC10763306; doi:10.1186/s12891-023-07149-4)
Supplement: Supplementary file 1 — Supplementary Material 1. Summary of patient information [file 12891_2023_7149_MOESM1_ESM.docx]

Table S1. Summary of patient information

| Patients | Sex | Age (Y) | BMI | Tear Size (cm) | Application |
| --- | --- | --- | --- | --- | --- |
| N-1 | Female | 65 | 21.5 | 2.0 | RNA-seq |
| N-2 | Female | 59 | 18.2 | 1.5 | RNA-seq |
| N-3 | Female | 70 | 28.8 | 1.5 | RNA-seq |
| N-4 | Male | 62 | 21.5 | 1.0 | qPCR |
| N-5 | Female | 66 | 23.6 | 2.5 | qPCR |
| N6 | Male | 55 | 22.8 | 2.0 | qPCR |
| DM-1 | Female | 63 | 24.2 | 1.5 | RNA-seq |
| DM-2 | Male | 58 | 25.9 | 2.0 | RNA-seq |
| DM-3 | Male | 52 | 27.7 | 2.0 | RNA-seq |
| DM-4 | Female | 68 | 28.9 | 1.5 | qPCR |
| DM5 | Female | 70 | 30.2 | 1.0 | qPCR |
| DM6 | Male | 60 | 25,7 | 2.0 | qPCR |
