# Supplementary material for: Analysis of differentially expressed genes in torn rotator cuff tendon tissues in diabetic patients through RNA-sequencing
Source: BMC Musculoskelet Disord. 2024 Jan 3;25:31. doi: 10.1186/s12891-023-07149-4 (PMC10763306; doi:10.1186/s12891-023-07149-4)
Supplement: Supplementary file 2 — Supplementary Material 2. Real-time quantitative PCR primer sequences [file 12891_2023_7149_MOESM2_ESM.docx]

**Table S2.** **Real‑time quantitative PCR primer sequences**

| **Target gene** | **Primers** | **Accession No** |
| --- | --- | --- |
| **Collagen 5** | F 5’- GAGAGGAGAACTGGGCTTCCAA-3’  R5’- TAGAGGTCCCACTTCTCCTGTC-3’ | NM_015719 |
| **MMP2** | F 5’- AGCGAGTGGATGCCGCCTTTAA-3’  R5’- CATTCCAGGCATCTGCGATGAG-3’ | NM_004530 |
| **EGR1** | F 5’- AGCAGCACCTTCAACCCTCAGG-3’  R5’- GAGTGGTTTGGCTGGGGTAACT-3’ | NM_001964 |
| **EGR2** | F 5’- CCTTTGACCAGATGAACGGAGTG-3’  R5’- GAAGGTCTGGTTTCTAGGTGCAG-3’ | NM_000399 |
| **GAPDH** | F 5’- GTCTCCTCTGACTTCAACAGCG-3’  R5’- ACCACCCTGTTGCTGTAGCCAA-3’ | NM_002046 |
