# Supplementary figures and images for: Analysis of differentially expressed genes in torn rotator cuff tendon tissues in diabetic patients through RNA-sequencing
Source: BMC Musculoskelet Disord. 2024 Jan 3;25:31. doi: 10.1186/s12891-023-07149-4 (PMC10763306; doi:10.1186/s12891-023-07149-4)

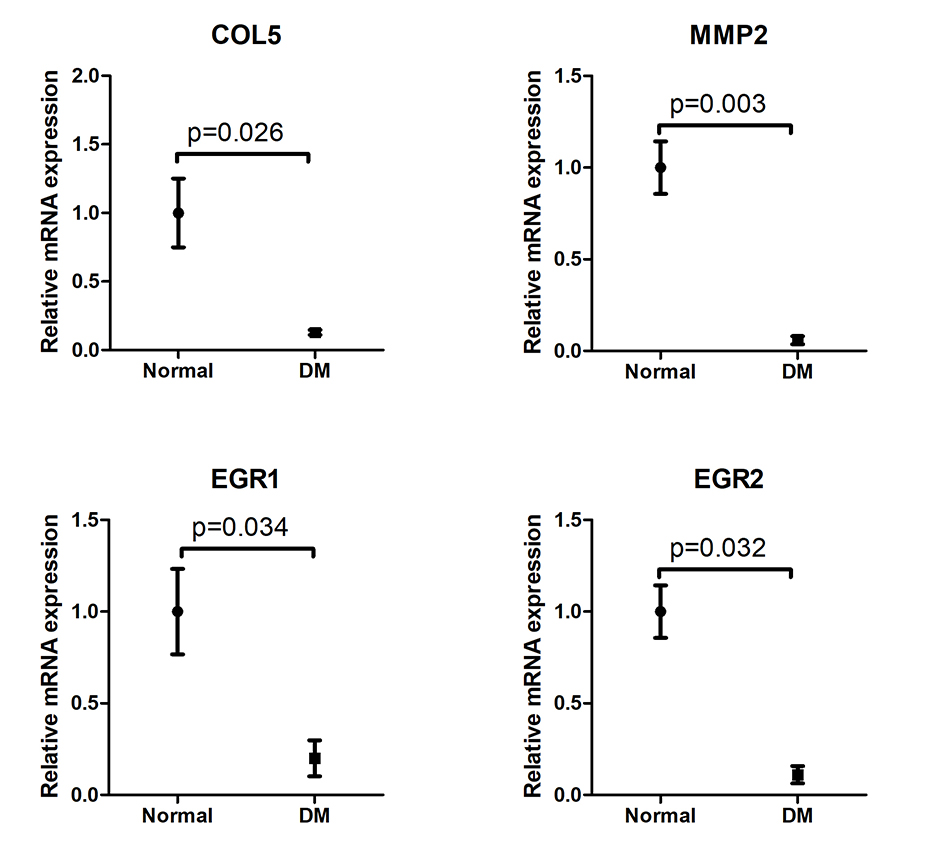

Supplement: Supplementary file 3 — Supplementary Material 3. Differentially expressed mRNAs [file 12891_2023_7149_MOESM3_ESM.jpg]
